# Increased liver regeneration rate and decreased liver function after synchronous liver and colon resection in rats

**DOI:** 10.1186/1750-1164-3-16

**Published:** 2009-12-24

**Authors:** Hideki Sasanuma, Frank Viborg Mortensen, Anders Riegels Knudsen, Peter Funch-Jensen, Masaki Okada, Hideo Nagai, Yoshikazu Yasuda

**Affiliations:** 1 Department of Surgery, Jichi Medical University, 3311-1, Shimotsuke, Tochigi 329-0498, Japan; 2 Department of Surgical Gastroenterology L, Aarhus University Hospital, Aarhus DK-8000, Denmark

## Abstract

**Background:**

The surgical strategy for the treatment of colorectal cancer and synchronous liver metastases remains controversial. The aim of the present study was to investigate the effects of colonic resection on liver function and regeneration in a rat model.

**Methods:**

Ninety-six Sprague-Dawley rats were block-randomized into six groups: Group I had a laparotomy performed. Group II had 1 cm colon resected and anastomosed. Group III and V had 40% or 70% of the liver resected, respectively. Additionally Group IV and VI had 1 cm colon resected and anastomosed, respectively. Body weight was recorded on postoperative day 0, 3, 5 and 7. Rats were sacrificed on postoperative day 7 by rapid collection of blood from the inferior vena cava, and endotoxin levels were measured. Remnant liver function was evaluated by means of branched amino acids to tyrosine ratio. Liver regeneration was calculated by (liver weight per 100 g of the body weight at sacrifice/preoperative projected liver weight per 100 g of the body weight) × 100.

**Results:**

The total number of complications was significantly higher in Group VI than Group I, III, IV, and V. Body weight and branched amino acids to tyrosine ratio were both significantly lower in rats that had simultaneous colonic and liver resection performed. Hepatic regeneration rate was significantly higher in the simultaneous colectomy group. Systemic endotoxin levels were unaffected by simultaneous colectomy on postoperative day 7.

**Conclusions:**

In our model morbidity seems to be related to the extent of hepatic resection. In rats undergoing liver resection, simultaneous colectomy induced a higher degree of hepatic regeneration rate. Body weight changes and branched amino acids to tyrosine ratio were negatively affected by simultaneous colectomy.

## Background

Surgical resection of colorectal liver metastases offers the best possibility for long-term survival. After complete resection of detectable metastases, about one third of patients survive for at least 5 years [1,2]. The surgical strategy for the treatment of colorectal cancer and synchronous liver metastases remains controversial. Simultaneous colorectal and liver resections might impose an increased risk of postoperative liver dysfunction and septic complications [3-5]. In a previous study, we showed that simultaneous hepatectomy was without effect on the healing of left sided colonic anastomoses, however, there was a tendency towards more postoperative complications [6]. The aim of the present study was to investigate the effects of colonic resection on liver function and regeneration in a rat model.

## Methods

### Animals

Ninety-six male Sprague-Dawley rats (body weight 200-250 g; Japan SLC, Inc., Hamamatsu, Shizuoka, Japan) were used for the experiments. Animals were acclimatised to our laboratory conditions for at least 7 days before the experiment and housed two or three each in stainless steel, wire-mesh cages. The animal room was maintained at a constant temperature and a 12-hour light-dark cycle. Unlimited access was allowed to standard laboratory food and tap water. This experiment was approved by the Ethics Committee of Jichi Medical School, Tochigi, Japan and the NIH Guide for the Care and Use of Laboratory Animals (Institute of Laboratory Animal Resources, National Research Council).

### Operative Procedure

The rats were block-randomised to one of 6 groups. Group I had a laparotomy performed. Group II had 1 cm of the left sided colon resected and anastomosed. Group III and V had 40% or 70% of the liver resected, respectively. Group IV and VI had 40% or 70% of the liver resected and 1 cm of the left sided colon resected and anastomosed, respectively. Our pilot study showed all rats (n = 14) undergoing over 80% liver resection died within 72 hours and, considering also the data by others, 70% liver resection was chosen as the largest tolerable liver resection. Bowel preparation, prophylactic and therapeutic antibiotics administration were not used. Under the diethyl ether anaesthesia, the abdominal wall hair was shaved with electric clippers, and the field was prepared with an alcohol solution. A laparotomy was performed through a 3.5-cm-long transverse incision. The liver resection was performed according to the principles described by Higgins and Anderson [7]. In rats undergoing 70% liver resection, the median lobe and left lateral lobe were removed. Left lateral lobe was removed in rats undergoing 40% liver resection. The pedicles of lobes were ligated with 5-0 sutures (Prolene^®^; Ethicon, Sommerville, New Jersey, USA), without portal clamping. Colorectal anastomoses were performed by means of eight interrupted 6-0 sutures (PDSII^®^; Ethicon). The abdominal wall was closed with 4-0 interrupted sutures in two layers (Maxon^®^; Davis & Geck, Wayne, New Jersey, USA). Autopsy was performed on all rats that died unexpectedly. Body weight was recorded on postoperative day (POD) 3, 5, and 7, and morbidity and mortality were recorded every day. On POD 7, the rats were anesthetised with diethyl ether, and a laparotomy was performed through the previous incision. The animals were inspected for signs of wound infection, and the abdominal cavity was examined for the presence of bowel obstruction, intraperitoneal abscesses, and anastomotic leakage. The rats were finally sacrificed by rapid collection of blood from the inferior vena cava. All procedures were performed by the same researcher.

### Liver Regeneration Rate

The preoperative total liver weight was calculated from resected liver weight in Group III, IV, V, and VI [7]. In Group I and II, we used 9.9 g as the preoperative total liver weight that was the mean value of Group III, IV, V, and VI. Postoperative total liver weight was measured at sacrifice. The change in liver weight in Group III, IV, V, and VI was evaluated as hepatic regeneration rate (RR). RR is defined as (liver weight per 100 g of the body weight at sacrifice/preoperative projected liver weight per 100 g of the body weight) × 100.

### Plasma Endotoxin Level and Branched-Chain Amino Acids to Tyrosine Ratio

Blood samples were taken from the inferior vena cava at the time of sacrifice. The concentration of plasma endotoxin was measured by turbidimetric time assay (Endotoxin-Single Test Wako^®^; Wako chemical, Tokyo, Japan), that measures the turbidity change in a coagulation reaction of a solution containing endotoxin and Limulus amoebocyte lysate [8]. The ratio of branched-chain amino acids to tyrosine (BTR) was measured by enzymatic method (Diacolor-BTR kit^®^; ONO Pharmaceutical, Osaka, Japan).

### Statistical Analysis

The effects of colectomy on plasma endotoxin level, liver regeneration rate, and BTR were analysed using analysis of variance (ANOVA) followed by Scheffé post hoc comparison. The changes in body weight through POD 7 were compared among the groups by repeated measures ANOVA. Differences in the total number of complications, including morbidity and mortality, between groups were tested for significance with the Kruskal-Wallis and multiple comparison tests. Probability values of less than 0.05 were considered to indicate statistical significance. All statistical analyses were performed with the statistical software package StatView^® ^for Windows (Version 5.0, SAS Institute Inc., Cary, North Carolina, USA).

## Results

### Mortality and Morbidity

Two rats, one in Group II and one in Group VI, had anastomotic leakage leading to the formation of intraperitoneal abscesses. In Group III, one rat developed intraperitoneal abscesses. In Group VI, one rat had bowel obstruction and wound infection, and another had wound infection only. The total number of complications was significantly higher in Group VI than in Group I, III, IV, and V (p ≤ 0.05, Table 1). Two rats in Group VI died; autopsies revealed no signs of wound infection, bowel obstruction, anastomotic leakage, or intraperitoneal abscesses.

**Table 1:** Influence of Colectomy (Cx) on Mortality and Morbidity in Rats Undergoing Different Extents of Hepatic Resection (Hx)

Groups(N = 96)	Mortality	Leakage	Obstruction	Abscess	Wound Infection	Total
Hx(-) + Cx(-) (Group I, n = 16)	0	-	-	0	0	0
Hx(-) + Cx (+) (Group II, n = 16)	0	1	0	1	0	2
Hx(40) + Cx(-) (Group III, n = 16)	0	-	-	1	0	1
Hx(40) + Cx(+) (Group IV, n = 16)	0	0	0	0	0	0
Hx(70) + Cx(-) (Group V, n = 16)	0	-	-	0	0	1
Hx(70) + Cx(+) (Group VI, n = 16)	2	1	1	1	2	7*

Hx(-): without hepatectomy, Hx(40): 40 percent hepatectomy, Hx(70): 70 percent hepatectomy, Cx(-): without colectomy, Cx(+): 1 cm colectomy. * p < 0.05 compared to groups I, III, IV, and V.

### Body Weight

**Figure 1:**
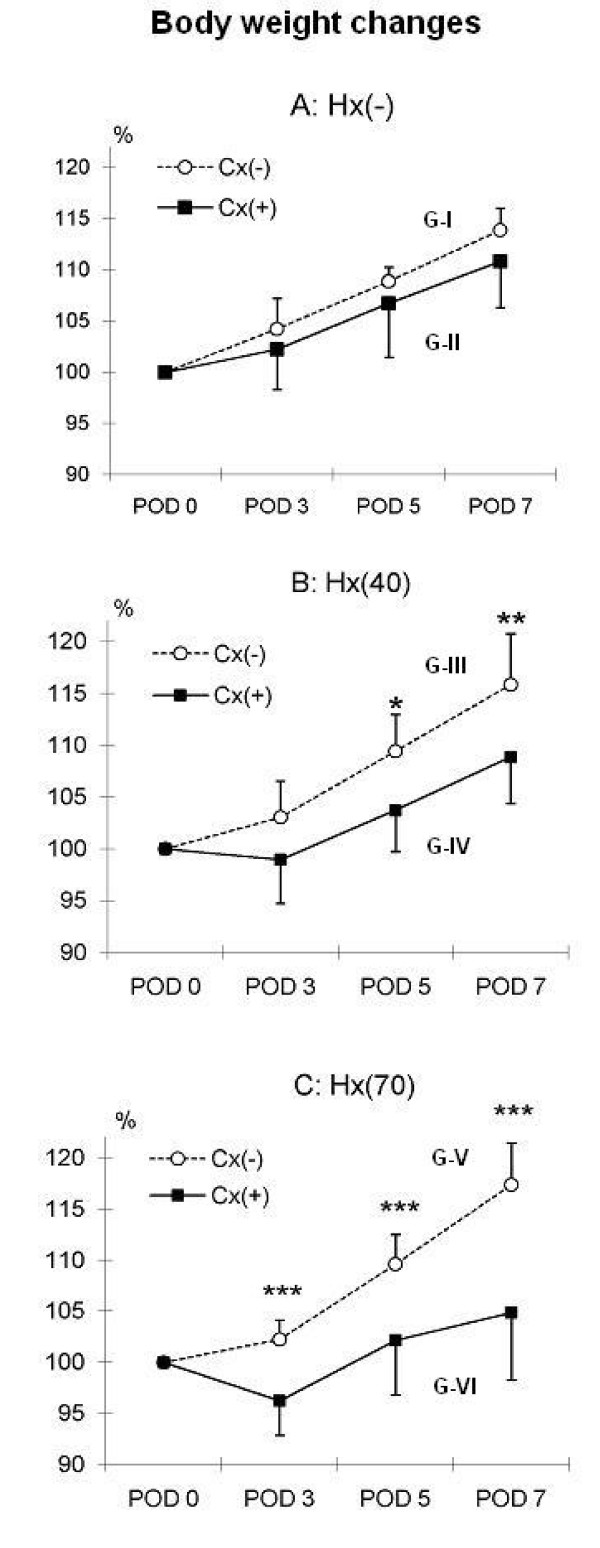
**Illustrating development in rat body weight compared to postoperative day (POD) 0 in percent**. Hx(-): without hepatectomy, Hx(40): 40 percent hepatectomy, Hx(70): 70 percent hepatectomy, Cx(-): without colectomy, Cx(+): 1 cm colectomy. G: Group 1A: No statistical significant difference in weight gain between the 2 groups was observed. 1B: At POD 5 and 7 rats without a simultaneous colectomy gained significantly more weight. 1C: At POD 3, 5, and 7 rats without a simultaneous colectomy gained significantly more weight, (*p ≤ 0.05, **p ≤ 0.01, and ***p ≤ 0.001).

Body weight changes were significantly affected by hepatectomy, Group I and II vs. Group III, IV, V and VI (p ≤ 0.05). In Group I vs. II no significant difference in body weight changes were found, Fig. 1A. Significant differences in body weight were observed on POD5 (p ≤ 0.05) and POD7 (p ≤ 0.01) in Group III vs. IV, Fig. 1B. In Group V vs. VI undergoing 70% hepatectomy, significant differences were found on any postoperative day (p ≤ 0.001), Fig. 1C.

### Branched-chain Amino Acids to Tyrosine Ratio

**Figure 2:**
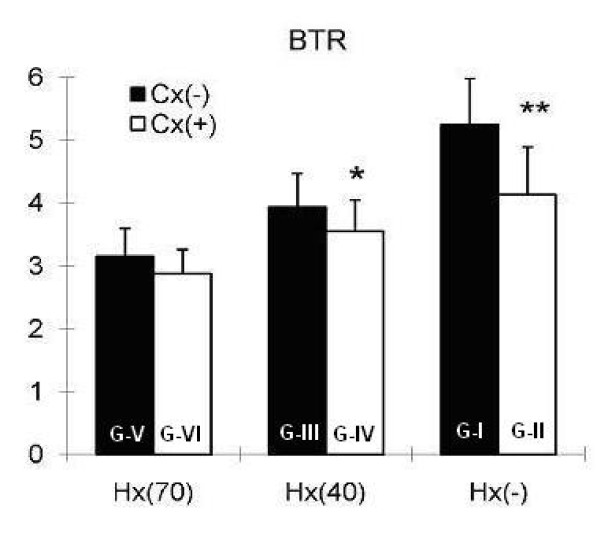
**Branched-chain Amino Acids to Tyrosine Ratio (BTR) in vena cava blood seven days after surgery**. Hx(-): without hepatectomy, Hx(40): 40 percent hepatectomy, Hx(70): 70 percent hepatectomy, Cx(-): without colectomy, Cx(+): 1 cm colectomy, G: Group, (*p ≤ 0.05 and **p ≤ 0.01).

Colectomy alone decreased BTR on POD 7 significantly in Group II compared to sham operated animals in Group I (p ≤ 0.001). After a 40% hepatectomy, BTR was significantly higher in rats not subjected to colectomy, Group III vs. IV (p ≤ 0.05). No significant difference was observed in BTR between Group V and VI (Fig. 2).

### Liver Regeneration Rate

**Figure 3:**
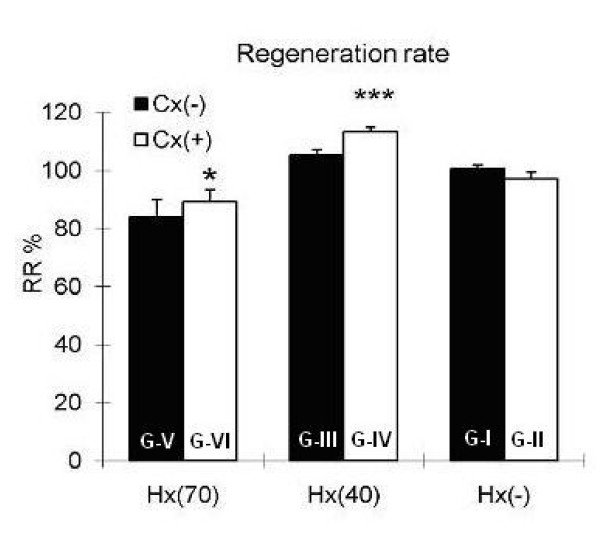
**Liver regeneration rate seven days after surgery**. Hx(-): without hepatectomy, Hx(40): 40 percent hepatectomy, Hx(70): 70 percent hepatectomy, Cx(-): without colectomy, Cx(+): 1 cm colectomy, G: Group, (*p ≤ 0.05 and ***p ≤ 0.001).

RR at POD 7 was significantly higher in rats subjected to simultaneous colectomy and hepatectomy (Group III vs. IV, p ≤ 0.001; Group V vs. VI, p ≤ 0.05). No significant difference was observed in RR between Groups I and II (Fig. 3).

### Serum Endotoxin Levels

**Figure 4:**
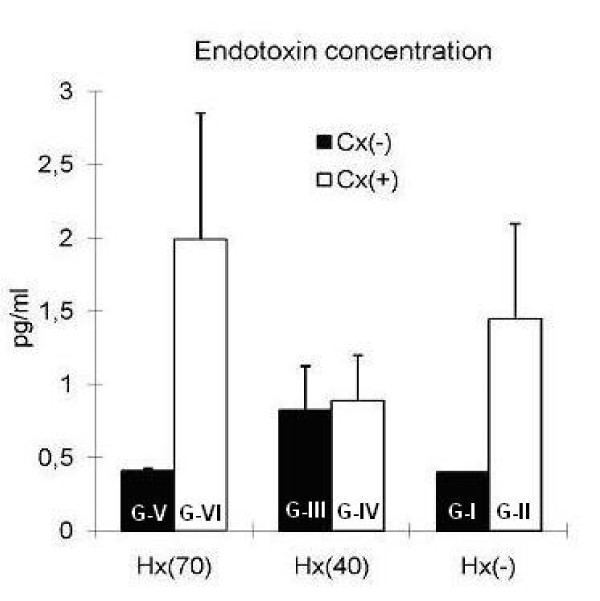
**Endotoxin concentration in vena cava blood seven days after surgery**. Hx(-): without hepatectomy, Hx(40): 40 percent hepatectomy, Hx(70): 70 percent hepatectomy, Cx(-): without colectomy, Cx(+): 1 cm colectomy. G: Group No statistical significant differences were found between the groups.

Serum endotoxin levels at POD 7 were not affected by colectomy (Fig. 4.)

## Discussion

The present study showed that rats that had a simultaneous hepatectomy and small colectomy performed had a significantly higher liver RR than rats that had only a hepatectomy carried out. BTR and weight development, on the other hand, were significantly lower in the simultaneous colectomy and hepatectomy groups on POD7. There were no significant differences in serum endotoxin levels between groups on POD 7. Excessive liver resection and colectomy could increase the risk of postoperative complications. Our results suggested simultaneous colon and liver resection has accelerated liver volume, however, liver function has not yet fully recovered at POD 7.

Endotoxin, i.e. the lipopolysaccharide (LPS) portion of the bacterial cell wall, is constantly produced in the gut from the death of Gram-negative bacteria. LPS is regularly absorbed into intestinal capillaries and a low grade of portal venous endotoxinemia is the normal status and an increase is seen during and after colonic surgery [9,10]. Endotoxins seem to be a promoter for liver regeneration [11,12]. Kupffer cells in the liver, representing the largest population of reticuloendothelial system macrophages, normally phagocytize and degrade all the LPS in the portal circulation [13]. Removing a large part of the liver reduces the total number of Kupffer cells and this, together with a higher load of LPS due to a colectomy, may lead to higher endotoxin levels in the systemic circulation [14,15]. We could not demonstrate a difference in endotoxin levels between groups on POD 7, partly due to a large variation. This of course does not rule out that there might have been differences earlier in the postoperative course.

Rats having a simultaneous hepatectomy and colectomy had a significantly lower weight gain than rats which only had a hepatectomy performed. Even though weight loss is a reliable marker of acute stress in animals, it is hard to accept this as the explanation for the difference we found as only 1 cm of the left colon was resected in the simultaneous groups and this represent only a slight additional trauma [16]. That the small colectomy only represents a slight additional trauma to the animals in the simultaneous groups is supported by the fact that we found no weight difference between rats that had only a laparotomy or colectomy performed. A more plausible explanation for the lower weight gain in the simultaneous groups could be a higher degree of endotoxinemia in the early postoperative course and this in combination with a liver resection might increase the surgical stress put on the animals.

The liver synthesizes its own proteins and several protein components of plasma and delivers a balanced mixture of amino acids to other organs via the blood. Removing a large part of the liver may, at least theoretically, impair delivery of amino acids. We choose BTR, a variable known to correlate with the Fisher ratio, serum albumin level, prothrombin time and retention of indocyanine green as a marker of liver function [17,18]. Rats having a simultaneous colectomy performed had a significantly lower BTR compared to rats that only had a hepatectomy carried out. As described above, it is hard to explain this finding with a bigger surgical trauma in the simultaneous groups as only 1 cm of the left colon was removed. A more plausible explanation for the lower BTR in the simultaneous groups could be a higher degree of systemic endotoxinemia in the early postoperative course as this has been shown to pronounce effects on the liver [19]. This is also supported by the fact that we found a significantly higher BTR in rats which had only a laparotomy performed compared to rats which had only a small colectomy carried out, i.e. these two groups are nearly identical with regard to the surgical stress put on them but one would expect a significantly higher degree of systemic endotoxinemia in the latter.

The liver is a crucial organ with high regenerative capacity and many complex functions. The liver is unique with respect to its anatomical position allowing continuous blood flow from the gastrointestinal tract through the sinusoids. The liver is also unique with respect to its cellular composition, comprising metabolically active hepatocytes, non-hepatocytic parenchymal cells, and various immune cells. By its location the liver, among other things, is allowed to function as a biochemical defense against toxic chemicals, hereunder endotoxins, entering from the gastrointestinal tract. Replacement of lost hepatic mass is mediated through proliferation of mature adult hepatocytes and the other hepatic cell types and not by proliferation of a selective subpopulation of stem cells [20]. In the present study we found a higher RR in rats which had a simultaneous hepatectomy and colectomy performed. This is in contrast to two other studies by Hachiya Y. et al. and Miyazaki M. et al. who both found an impaired liver regeneration after simultaneous bowel resection in rats [21,22]. Their set-up differed however from ours in that their bowel resections were bigger, i.e. they exposed the animals to greater surgical stress than we did and this might have implications for liver regeneration. In fact the colon resection in our study was so small; the numbers of complications were smaller, that our model might be regarded as a way to achieve endogenous endotoxinemia without exposing animals to much additional surgical stress. The higher RR in the simultaneous groups could be explained by a higher amount of systemic endotoxinemia as these compounds, as described earlier, have been shown to stimulate liver regeneration [11,12]. In the studies by Hachiya Y. et al. and Miyazaki M. et al. the hepatothrophic effect of the increased amount of endotoxins in the portal blood in their simultaneous groups might have been overwhelmed by a negative hepatothrophic effect of the greater surgical trauma [21,22].

In conclusion, when planning simultaneous colorectal and extended liver resections one should be aware that this procedure, although stimulating the liver's regenerative capacity judged by RR, seems to restrict liver function judged by BTR. In our model morbidity seems to be related to the extent of hepatic resection. Further studies on humans are needed to investigate the clinical implications of these findings.

## Abbreviations

POD: Postoperative day; RR: Regeneration rate; BTR: Branched-chain Amino Acids to Tyrosine Ratio; ANOVA: Analysed of variance; LPS: Lipopolysaccharide

## Competing interests

The authors declare that they have no competing interests.

## Authors' contributions

HS carried out all rat studies and drafted the manuscript. FVM and ARK participated in the design of the study and performed the statistical analysis. FVM and YY conceived of the study, and participated in its design and coordination and PFJ, MO, and HN helped to draft the manuscript. All authors read and approved the final manuscript.
